# Supplementation with D-serine prevents the onset of cognitive deficits in adult offspring after maternal immune activation

**DOI:** 10.1038/srep37261

**Published:** 2016-11-17

**Authors:** Yuko Fujita, Tamaki Ishima, Kenji Hashimoto

**Affiliations:** 1 Division of Clinical Neuroscience, Chiba University Center for Forensic Mental Health, Chiba 260-8670, Japan

## Abstract

Prenatal maternal infection contributes to the etiology of schizophrenia, with D-serine, an endogenous co-agonist of the *N*-methyl-D-aspartate (NMDA) receptor, playing a role in the pathophysiology of this disease. We examined whether supplementation with D-serine during juvenile and adolescent stages could prevent the onset of cognitive deficits, prodromal and the core symptoms of schizophrenia in adult offspring after maternal immune activation (MIA). Juvenile offspring exposed prenatally to poly(I:C) showed reduced expression of NMDA receptor subunits in the hippocampus. Supplementing drinking water with D-serine (600 mg/L from P28 to P56) prevented the onset of cognitive deficits in adult offspring after MIA, in a significant manner. This study shows that supplementing offspring with D-serine during juvenile and adolescent stages could prevent the onset of psychosis in adulthood, after MIA. Therefore, early intervention with D-serine may prevent the occurrence of psychosis in high-risk subjects.

Multiple lines of evidence suggest that hypofunction of glutamatergic neurotransmission via the *N*-methyl-D-aspartate (NMDA) receptor plays a crucial role in the pathophysiology of schizophrenia^1^,^2^,^3^,^4^,^5^,^6^,^7^,^8^,^9^,^10^,^11^. D-serine, an obligatory co-agonist at the NMDA receptor, is integral to neurotransmission via NMDA signaling throughout development and into adulthood^12^,^13^. A number of clinical studies have highlighted disturbed NMDA receptor neurotransmission due to decreased D-serine levels as a causative factor in the pathophysiology of schizophrenia^4^,^5^,^6^,^7^,^8^,^9^,^14^,^15^,^16^,^17^. First, there are reports showing lower levels of D-serine in the blood, cerebrospinal fluid (CSF), and postmortem brain tissue from patients with schizophrenia, relative to normal controls^18^,^19^,^20^,^21^,^22^,^23^. Secondly, treatment with D-serine is beneficial for alleviating several symptoms associated with schizophrenia^24^,^25^,^26^, even in treatment-resistant disease^27^,^28^. Meta-analyses support these findings that D-serine is effective in treating schizophrenia^29^,^30^, although D-serine is not approved as therapeutic drug for schizophrenia. Thirdly, mRNA expression and the activity of D-amino acid oxidase (DAAO), which metabolizes D-serine, is increased in postmortem brains of schizophrenic patients^21^,^31^,^32^. Endogenous D-serine is synthesized from L-serine by serine racemase (SRR)^33^. Levels of SRR protein in the prefrontal cortex and hippocampus of schizophrenia cohorts were lower than those of control groups^21^. Finally, the G72 gene located at chromosome 13q is significantly associated with schizophrenia^34^,^35^. This gene has been designated a DAAO activator, since the G72 protein interacts physically with DAAO^34^. Meta-analyses provided evidence of significant association between G72/G30 genes and schizophrenia^35^,^36^,^37^. Interestingly, there are two reports showing increased G72 protein levels in the blood of patients with schizophrenia^38^,^39^. A subsequent largest GWAS study of schizophrenia demonstrated the *SRR* gene as a susceptible gene^40^.

Multiple epidemiological studies support the neurodevelopmental hypothesis for the pathogenesis of schizophrenia^41^. Maternal immune activation (MIA) is an environmental risk factor for the development of psychiatric disorders, such as schizophrenia, and a prenatal immune challenge by the viral mimetic, poly(I:C) is capable of inducing long-lasting behavioral abnormalities in adulthood^42^,^43^,^44^,^45^. It is thought that prenatal poly(I:C) exposure attenuated the expression of GluN1, a subtype of the NMDA receptor in the brains of P21 rat offspring^45^, implicating NMDA receptor hypofunction in juvenile offspring after MIA. These findings point to the possibility that hypofunction at this receptor in juvenile offspring after MIA could interfere with normal fetal brain neurodevelopment, and that these deficits promote the onset of schizophrenia in adulthood.

Cognitive impairment is detectable in subjects at high-risk for psychosis several years preceding onset of frank disease^46^,^47^. Interestingly, high-risk subjects who later developed psychosis showed poorer neurocognitive functioning compared with those who did not develop a psychotic disorder^47^, indicating that cognitive impairment could be a risk factor for conversion to psychosis. It is clear that providing early intervention at the prodromal phase of psychosis is one of the most important and challenging tasks in psychiatry. This study was undertaken to examine whether D-serine supplementation from juvenile stages (P28) to adolescence (P56) could prevent the onset of cognitive deficits in adult offspring (<P70), after MIA.

## Results

### Cognitive deficits in juvenile offspring after MIA

**Figure 1:**
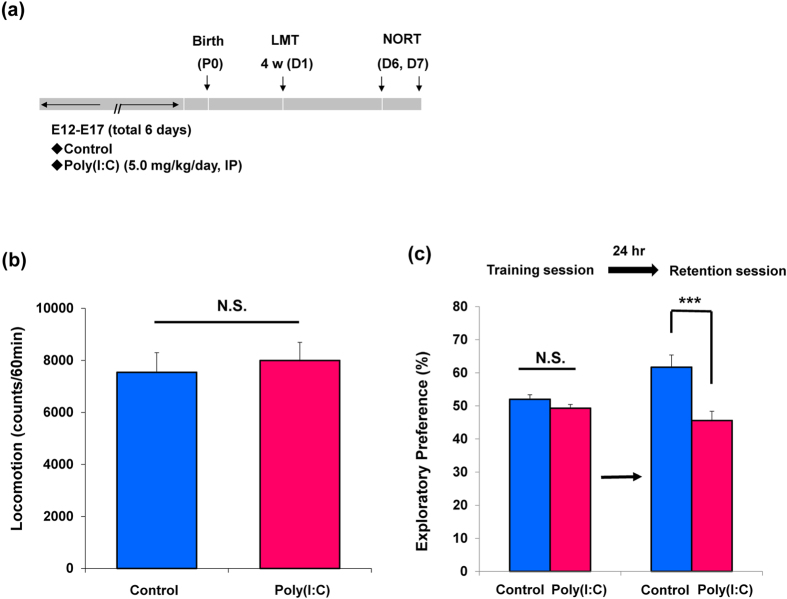
Behaviors in the juvenile offspring after prenatal poly(I:C) exposure. (**a**): Schedule of treatment and behavioral tests. Saline or poly(I:C)(5 mg/kg/day from E12 to E17) was injected into pregnant mice. Behavioral tests including locomotion (LMT: D1) and novel object recognition test (NORT: D6 and D7) were performed. (**b**): Locomotion: There was no difference between ploy(I:C) offspring group and control group at juvenile stage. The value is expressed as the mean ± SEM. (n = 13 for control group, n = 19 for poly(I:C) group). (**c**): Novel object recognition test (NORT): the exploratory preferences were significantly lower in the poly(I:C) offspring than controls in the retention session, but there was no difference between the two groups in the training session. ***P < 0.001 compared with control group. The value is expressed as the mean ± SEM (n = 13 for control group, n = 18 for poly(I:C) group).

Behavioral tests of juvenile offspring were performed during P28-P35 after prenatal poly(I:C)(5 mg/kg/day from E12 to E17) injections (Fig. 1a). In the open field test, spontaneous locomotion was unchanged (P = 0.670) between control group and poly(I:C)-treated group (Fig. 1b). In the novel object recognition test (NORT), there was no difference (P = 0.141) between two groups in the training session. However, in the retention session, the exploratory preference of poly(I:C) group was significantly (P = 0.001) lower than that of control (Fig. 1c). These results imply that prenatal poly(I:C) exposure induces cognitive deficits in juvenile offspring.

### Levels of amino acids and their ratios in the brain regions of juvenile offspring after MIA

We measured tissue levels of amino acids (glutamate, glutamine, glycine, L-serine, D-serine, γ-amino butylic acid (GABA)) in the frontal cortex, hippocampus, and striatum at juvenile stage (P28). Treatment with poly(I:C) significantly increased levels of glutamate and glutamine in the frontal cortex, but significantly decreased levels of GABA in the frontal cortex (Table 1). Furthermore, treatment with poly(I:C) significantly decreased levels of glutamate in the hippocampus, but significantly increased levels of glycine in the hippocampus (Table 1). Moreover, treatment with poly(I:C) significantly increased levels of glycine and L-serine in the striatum, whereas other amino acids were not altered (Table 1). Levels of D-serine in the three regions remained the same (Table 1).

The ratio of glutamine to glutamate in the hippocampus of poly(I:C) group was significantly lower than that of control group, suggesting abnormalities in glutamine-glutamate cycle in the hippocampus of juvenile offspring after prenatal poly(I:C) injections (Table 1). Furthermore, the ratio of D-serine to L-serine in the frontal cortex and striatum of poly(I:C) group was significantly lower than that of control group, suggesting reduced production of D-serine from L-serine in these regions (Table 1). Moreover, the ratio of GABA to glutamate in the frontal cortex of poly(I:C) group was significantly lower than that of control group whereas this ratio in the hippocampus of poly(I:C) group was slightly higher than that of control group (Table 1). These findings suggest abnormalities in the NMDA receptor neurotransmission in the brain of juvenile offspring after MIA.

### Alterations in the gene expression of SRR, DAO, and NMDA receptor subunits in the brain from juvenile offspring after MIA

**Figure 2:**
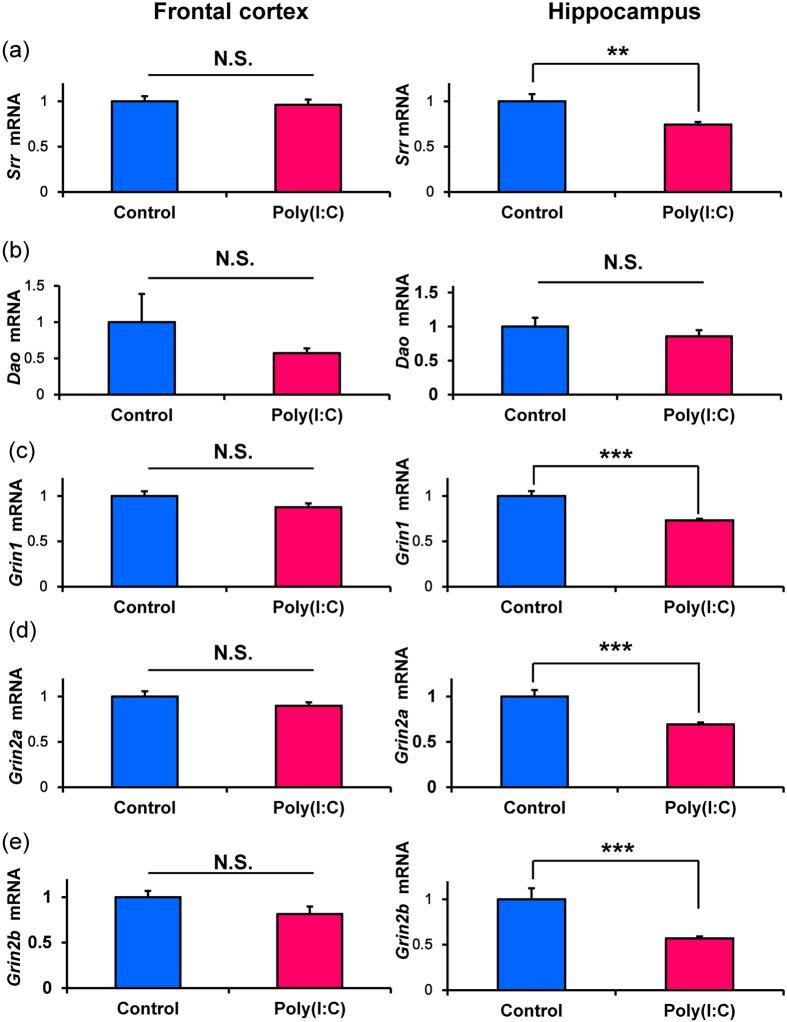
Gene expression in the frontal cortex and hippocampus from the juvenile offspring after prenatal poly(I:C) exposure. (**a**): Serine racemase (*Srr*). (**b**): D-amino acid oxidase (*Dao*). (**c**): GluN1 subtype of the NMDA receptor (*Grin1*). (**d**): GluN2A subtype of NMDA receptor (*Grin2a*). (**e**): GluN2B subtype of the NMDA receptor (*Grin2b*). Data represent the mean ± S.E.M. (n = 10 for control group, n = 14 for poly(I:C) group). *P < 0.05, **P < 0.01, *** P < 0.001 compared with control group.

We measured gene expression of serine racemase (*Srr*), DAO (*Dao*), and the NMDA receptor subunits (*Grin1*, *Grin2a*, *Grin2b*) in the frontal cortex and hippocampus. Expression of *Srr* in the hippocampus of poly(I:C) group was significantly (P = 0.002) lower than that of control group although expression of *Srr* in the frontal cortex was not different (Fig. 2a). Furthermore, expression of *Dao* in the PFC and hippocampus was not different (P = 0.357) for two groups (Fig. 2b). Expressions of *Grin1* (P < 0.001), *Grin2a* (P < 0.001), and *Grin2b* (P < 0.001) in the hippocampus of poly(I:C) group were significantly lower than those of control group (Fig. 2c–e). In contrast, expressions of *Grin1*, *Grin2a*, and *Grin2b* in the frontal cortex were not different for two groups (Fig. 2c–e). These findings suggest alterations in the NMDA receptor function in the hippocampus of juvenile offspring after prenatal poly(I:C) injections.

### Cognitive deficits in adult offspring after MIA

**Figure 3:**
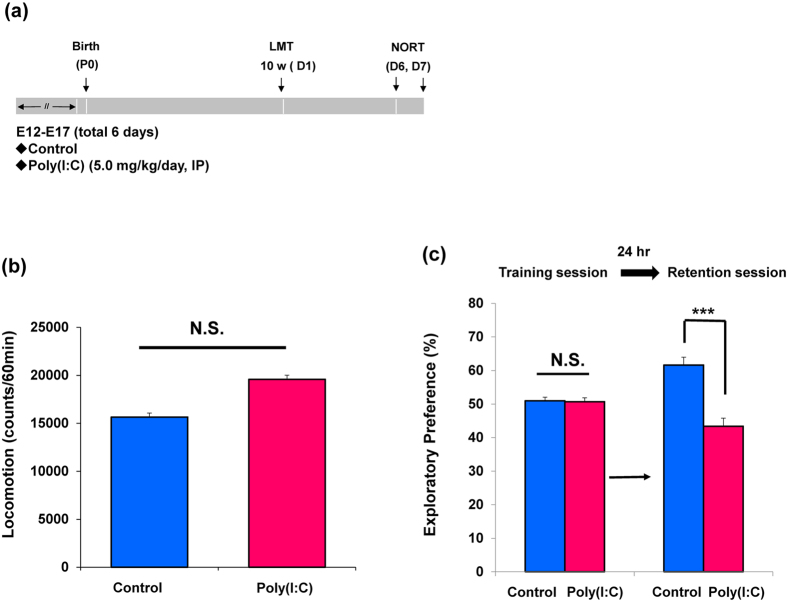
Behaviors in the adult offspring after prenatal poly(I:C) exposure. (**a**): Schedule of treatment and behavioral tests. Saline or poly(I:C)(5 mg/kg/day from E12 to E17) was injected into pregnant mice. Behavioral tests including locomotion (LMT: 10 W (D1)) and novel object recognition test (NORT: D6 and D7) were performed. (**b**): Locomotion: There was no difference between ploy(I:C) offspring and controls at juvenile stage. The value is expressed as the mean ± SEM. (n = 21). (**c**): NORT: The exploratory preferences were significantly lower in the poly(I:C) offspring than controls in the retention session, but there was no difference between the two groups in the training session. ***P < 0.001 compared with control group. The value is expressed as the mean ± SEM (n = 22 for control group, n = 20 for poly(I:C) group).

Behavioral tests of juvenile offspring were performed during P70-P84 after prenatal poly(I:C)(5 mg/kg/day from E12 to E17) injections (Fig. 3a). In the open field test, locomotion was significantly unchanged (P = 0.088) between two groups (Fig. 3b). In the NORT, there was no difference (P = 0.850) between two groups in the training session. However, in the retention session, the exploratory preference of poly(I:C) group was significantly (P < 0.001) lower than that of control (Fig. 3c). These findings indicate that prenatal poly(I:C) exposure caused cognitive in adult offspring.

### Levels of amino acids and their ratios in the brain regions of adult offspring after MIA

Treatment with poly(I:C) significantly decreased levels of D-serine in the frontal cortex, whereas other amino acids were not altered (Table 2). Furthermore, treatment with poly(I:C) significantly decreased levels of glutamate, L-serine, and D-serine in the hippocampus (Table 2). Moreover, treatment with poly(I:C) significantly decreased levels of L-serine, and D-serine in the striatum whereas glycine levels were increased in the poly(I:C) group (Table 2). Interestingly, levels of D-serine in the three regions were significantly lower than those of control group (Table 2).

The ratio of glutamine to glutamate in the three regions was not different (Table 2). Furthermore, the ratio of L-serine to glycine in the hippocampus and striatum of poly(I:C) group was significantly lower than that of control group, suggesting alterations in the L-serine – glycine conversion in these regions (Table 2). Moreover, the ratio of D-serine to L-serine in the frontal cortex and hippocampus of poly(I:C) group was significantly lower than that of control group, suggesting alterations in the D-serine – L-serine conversion in these regions (Table 2). The ratio of GABA to glutamate in the hippocampus of poly(I:C) group was significantly higher than that of control group (Table 2). These findings suggest abnormalities in the NMDA receptor neurotransmission in the brain regions of adult offspring after MIA.

### Supplementation of D-serine in drinking water prevents cognitive deficits in adult offspring after MIA

**Figure 4:**
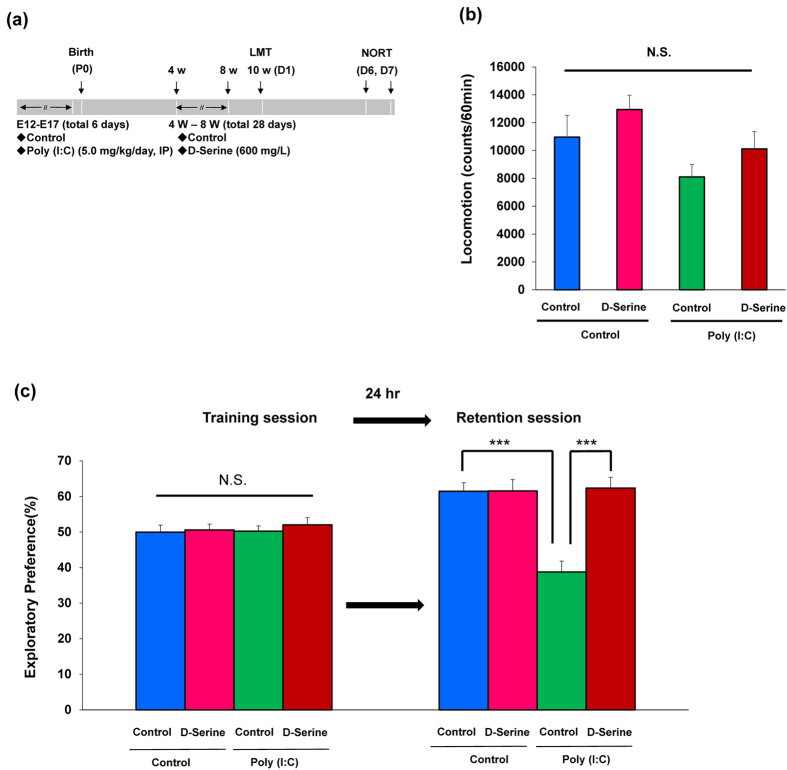
Effects of D-serine supplementation on cognitive deficits in the adult offspring after prenatal poly(I:C) exposure. (**a**): Schedule of treatment and behavioral tests. Saline or poly(I:C)(5 mg/kg/day from E12 to E17) was injected into pregnant mice. Vehicle or D-serine (600 mg/L) in drinking water was given into mice from 4-week to 8-week olds. Behavioral tests including locomotion (LMT: 10 W (D1)) and novel object recognition test (NORT: D6 and D7) were performed. (**b**): Locomotion: there was no significant difference among the four groups in the locomotor activity. The value is expressed as the mean ± SEM (n = 10–15). N.S.: not significant. (**c**): NORT: The exploratory preferences were significantly lower in the poly(I:C) offspring than controls in the retention session, but there was no difference between the two groups in the training session. ***P < 0.001 compared with control group. The value is expressed as the mean ± SEM (n = 9–13).

We examined whether D-serine was capable of preventing cognitive deficits in adult offspring after MIA. From P28 to P56, D-serine (600 mg/L) or a vehicle in drinking water was given into mice. To exclude the acute effects of D-serine, water in drinking water was given into all mice for 2-weeks (from P57 to P70) before behavioral tests (from P70 to P84)(Fig. 4a). Two-way ANOVA of locomotion data revealed no difference (poly(I:C): F_1,43_ = 5.467, P = 0.024, D-serine: F_1,43_ = 2.698, P = 0.108, Interaction: F_1,43_ = 0.000, P = 0.987) among the four groups (Fig. 4b). In the training session of NORT, there was no difference (poly(I:C): F_1,42_ = 0.230, P = 0.634, D-serine: F_1,42_ = 0.450, P = 0.506, Interaction: F_1,42_ = 0.110, P = 0.742) between four groups (Fig. 4c). In the retention session, two-way ANOVA of NORT data revealed statistical significance (poly(I:C): F_1,42_ = 13.58, P = 0.001, D-serine: F_1,42_ = 15.83, P < 0.001, Interaction: F_1,42_ = 15.66, P < 0.001) among the four groups (Fig. 4c). The exploratory preference of poly(I:C) group was significantly lower than that of control, and supplementation of D-serine significantly improved poly(I:C)-induced cognitive deficits in adult offspring (Fig. 4c).

## Discussion

**Figure 5:**
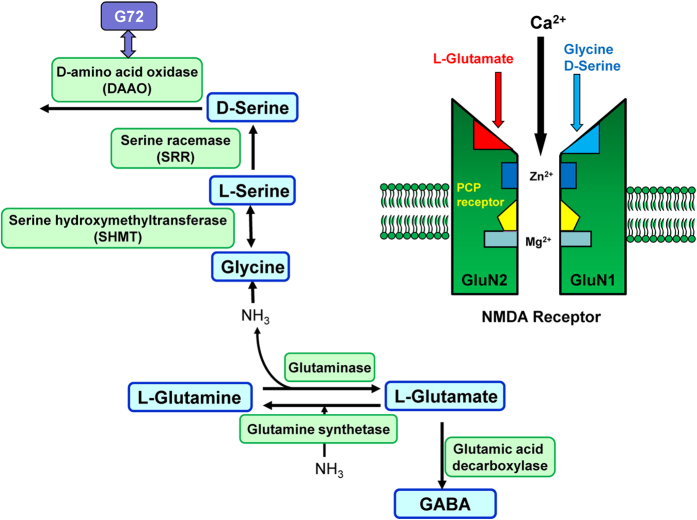
Synthetic and metabolic pathway of amino acids and NMDA receptor. L-Glutamate, an excitatory amino acid, is synthesized from L-glutamine by glutaminase, and metabolized to L-glutamine by glutamine synthetase. In addition, L-glutamate is metabolized to γ-aminobutyric acid (GABA), an inhibitory amino acid, by glutamic acid decarboxylase. D-Serine is synthesized from L-serine by serine racemase (SRR), and is metabolized by D-amino acid oxidase (DAAO). L-Serine is converted to glycine by serine hydroxymethyltransferase (SHMT). Phencyclidine (PCP) is an ion-channel blocker of the NMDA receptor. Glycine and D-serine are endogenous co-agonists of the glycine modulatory site on the GluN1 subunit. Glutamate is an endogenous agonist at glutamate sites on the GluN2 subunit. Thus, glutamine-glutamate-GABA cycle plays a key role in the NMDA receptor neurotransmission. (A slight modification with ref. 8).

In this study, we found that prenatal exposure to poly(I:C) caused cognitive deficits in juvenile and adult offspring. Furthermore, it also caused alterations in the levels and the ratio of crucial amino acids (glutamate, glutamine, glycine, D-serine, L-serine, GABA) in the brains of juvenile and adult offspring. These amino acids are related to the glutamine-glutamate-GABA cycle in the brain^7^,^8^,^48^(Fig. 5). Moreover, gene expression of *Srr*, *Grin1*, *Grin2a*, and *Grin2b* in the hippocampus of poly(I:C) treated animals was significantly lower than that of control groups, suggesting NMDA receptor hypofunction in the hippocampus of juvenile offspring after MIA. Finally, supplementation with D-serine during juvenile and adolescent stages could prevent cognitive deficits in adult offspring after MIA. Considering the crucial role of NMDA receptors in brain development, it is likely that prenatal poly(I:C) exposure causes NMDA receptor hypofunction in the brains of juvenile offspring, giving rise to the later life behavioral abnormalities seen in adult offspring after MIA. It is therefore possible that treatment with D-serine could prevent the onset of psychosis in high-risk subjects.

We found reduced expression of the *Srr* gene in the hippocampus of juvenile offspring after prenatal poly(I:C) exposure, although levels of D-serine and L-serine and the ratio of D- to L-serine in the hippocampus remained the same. We also found reduced gene expression of NMDA receptor subtypes,*Grin1*, *Grin2a*, and *Grin2b* in the hippocampus of juvenile offspring after prenatal poly(I:C) exposure. Thus, it seems that disturbance of NMDA receptor function in the hippocampus might play a role in the cognitive deficits seen in juvenile offspring after MIA. It was shown that prenatal poly(I:C)(10 mg/kg/day on days E14, E16 and E18) exposure caused a reduction of *Grin1* in rat brains from P21 offspring^45^. Other research suggested that prenatal poly(I:C) (5 mg/kg on gestation day 17) exposure significantly reduced GluN1 protein levels in the dorsal hippocampus of adult offspring^49^. Taken together, it is likely that maternal activation of the immune system can interfere with NMDA receptor function during brain development, inducing cognitive deficits in juvenile and adult offspring. Further detailed studies on how prenatal poly(I:C) exposure induces the NMDA receptor hypofunction and behavioral abnormalities in juvenile and adulthood are needed.

In this study, we found significant alterations in the D-serine levels in three brain regions of adult offspring after MIA although D-serine levels were not altered in juvenile offspring, indicating neurodevelopmental changes of D-serine in the poly(I:C) model. Furthermore, we found significant alterations in GABA levels and GABA/glutamate ratio in the frontal cortex from juvenile offspring after MIA although these findings were recovered to control levels at adult offspring after MIA. Together, these findings suggest neurodevelopmental changes in the synthesis and metabolism of amino acids in the brain regions after MIA.

Patients with schizophrenia show non-psychotic and non-specific prodromal symptoms, such as cognitive impairment, for several years preceding the onset of frank psychosis^46^,^47^. A meta-analysis of 27 studies showed that the average rate of transition to full psychosis among such patients is 22 percent within the first year and 36 percent within three years^47^. Therefore, providing early intervention at the prodromal phase of schizophrenia and related psychosis is one of the most important and challenging tasks in psychiatry^50^. Here, we found that prenatal poly(I:C) exposure induced cognitive deficits in juvenile offspring, suggesting that these offspring may show prodromal, or at risk of psychosis symptoms. Interestingly, we found that supplementation with D-serine from juvenile to adolescent stages prevented cognitive deficits in adult offspring after MIA. Previously, we also reported that chronic administration of D-serine (900 mg/kg/day from P35 to P70) significantly prevented the onset of behavioral abnormalities after neonatal exposure to phenazine methosulfate (a SRR inhibitor)^51^. Very interestingly, a recent double-blind, placebo-controlled, randomized study showed that D-serine (60 mg/kg/day for 16 weeks) could prevent the conversion to psychosis in individuals at clinical high risk of schizophrenia^52^. These findings make D-serine an attractive prophylactic amino acid for early intervention in the onset of schizophrenia^53^, mainly because D-serine is effective for treating several symptoms in schizophrenia^24^,^25^,^26^,^27^,^28^,^29^,^52^.

In conclusion, our results suggest that prenatal poly(I:C) exposure causes cognitive deficits relevant to prodromal symptoms, during juvenile and adult stages. Interestingly, supplementation with D-serine from juvenile to adolescent stages could prevent cognitive deficits in adult offspring after MIA, indicating that D-serine may serve as an early intervention for psychosis.

## Methods and Materials

### Animals

Pregnant ddY mice (E5, 9–10 weeks old) were purchased from Japan SLC Inc. (Hamamatsu, Shizuoka, Japan). The mice were housed in clear polycarbonate cages (22.5 × 33.8 × 14.0 cm), under a controlled 12/12 hour light-dark cycle (lights on from 07:00 am to 07:00 pm), with room temperature at 23 ± 1 °C and humidity at 55 ± 5%. The mice were given free access to water and food pellets. All experiments were carried out in accordance with the Guide for Animal Experimentation of Chiba University. The protocol was approved by the Chiba University Institutional Animal Care and Use Committee.

### Prenatal administration of poly(I:C)

Treatment schedule of poly(I:C) was performed according to our previous reports^42^,^54^. Every six consecutive days from E12 to E17, the pregnant mice were injected intraperitoneally (i.p.) with poly(I:C)(5.0 mg/kg, Sigma-Aldrich Co. Ltd., USA) dissolved in physiological saline, or an equivalent volume of saline. The male mice of offspring were separated from their mothers after 3 weeks, and mice were caged in separate groups.

### Supplementation of D-serine as drinking water

To examine whether D-serine supplementation during juvenile and adolescence could prevent the onset of behavioral abnormalities in adult mice of offspring after MIA, D-serine (600 mg/L, Sigma-Aldrich, St. Louis, MO, USA) or vehicle (water) were administered as drinking water from P28 to P56; this period is thought to represent juvenile to adolescence. The dose resulted in a daily dose of approximately 100 mg/kg D-serine per body weight (average weight: 30 g, average drinking volume: 5 mL/day). From P57, all mice received water. Behavioral tests were performed at adulthood (P70-P84).

### Measurement of amino acids in the brain

At juvenile (P28), and adult (P70) stages, mice were sacrificed, and their brains were removed for measurement of amino acids. The frontal cortex, hippocampus and striatum were quickly dissected on ice from whole brain. The dissected tissues were weighed and stored at −80°C until assayed.

Briefly, brain tissues were homogenized in 1.5 mL of methanol (HPLC grade) on ice. The homogenates were centrifuged at 3000 g for 6 min at 4 °C, and 20 μL of supernatant was evaporated to dryness at 40 °C. To the residue, 20 μL of H_2_O (HPLC grade), 20 μL of 0.1 M borate buffer (pH 8.0), and 60 μL of 50 mM 4-fluoro-7-nitro-2,1,3-benzoxadiazole (NBD-F; Tokyo Kasei Kogyo Co., Ltd., Tokyo, Japan) in CH_3_CN (HPLC grade) were added. The reaction mixture was then heated to 60 °C for 2 min, and immediately supplemented with 100 μL of H_2_O/acetonitrile (90/10) containing 0.1% trifluoroacetic acid (TFA) to stop the reaction. Levels of amino acids (D-serine, L-serine, glycine, glutamine, glutamate, GABA) were measured using high performance liquid chromatography (HPLC) system (Shimadzu Corporation, Kyoto, Japan), as previously reported^48^,^55^. Fluorescence detection was performed at 530 nm with an excitation wavelength of 470 nm.

### Measurement of gene expression in the brain

At juvenile (P28) stage, mice were sacrificed, and their brains were removed for measurement of gene expression of *Srr*, *Dao*, *Grin1*, *Grin2a*, and *Grin2b*. The frontal cortex and hippocampus were quickly dissected on ice from whole brain. A quantitative RT-PCR system (Step One Plus, Thermo Fisher Scientific, Yokohama, Japan) was used to measure mRNAs. The specific mRNA transcripts were quantified by TaqManGene Expression assays (Thermo Fisher Scientific, Yokohama, Japan). Expression levels of *Srr* (Mm00489123_m1), *Dao* (Mm00438378_m1), *Grin1* (Mm00433790_m1), *Grin2a* (Mm00433802_m1), and *Grin2b* (Mm00433820_m1) were measured in brain tissue. Total RNA was extracted by use of an RNeasy Mini Kit (Qiagen, Hilden, Germany). The purity of total RNA was assessed by Biophotometer plus (Eppendorf, Hamburg, Germany). the RNA samples were used in the first strand cDNA synthesis with High Capacity cDNA Reverse Transcription Kit (#4368813 Thermo Fisher Scientific, Yokohama, Japan). All samples were tested in triplicate and average values were used for quantification. The average values were normalized to Vic-labeled *Actb* mRNA (#4352341E: pre-developed TaqMan Assay Reagents, Thermo Fisher Scientific, Yokohama, Japan).

### Locomotor activity in mice

Both horizontal and rearing activity were monitored by an infrared ray passive sensor system (SCANET-SV10, Melquest Ltd, Toyama, Japan), and activity was integrated every 10 minutes, as previously reported^51^,^54^,^56^. Individual mice were placed in activity chambers and allowed 2 hours of free exploration as spontaneous activity.

### Novel object recognition test (NORT)

The NORT was performed as previously reported^51^,^54^,^57^,^58^. Before testing, mice were habituated in the box for 3 days. During a training session, two objects (differing in shape and color but of similar size) were placed in the box 35.5 cm apart (symmetrically), and each animal was allowed to explore in the box for 5 minutes. The animals were considered to be exploring the object when the head of the animal was both facing and within 2.54 cm of the object or when any part of the body, except for the tail was touching the object. The time that mice spent exploring each object was recorded. After training, mice were immediately returned to their home cages, and the box and objects were cleaned with 75% ethanol, to avoid any possible instinctive odorant cues. Retention tests were carried out at one-day intervals, following the respective training. During the retention test, each mouse was reintroduced into their original test box, and one of the training objects was replaced by a novel object. The mice were then allowed to explore freely for 5 minutes, and the time spent exploring each object was recorded. Throughout the experiments, the objects were counter-balanced, in terms of their physical complexity and emotional neutrality. A preference index, that is, the ratio of time spent exploring either of the two objects (training session) or the novel object (retention test session) over the total time spent exploring both objects, was used.

### Statistical analysis

All data are shown as mean ± standard error of the mean (S.E.M.). The data of amino acids, locomotion, and NORT were analyzed by Student’s t-test, or two-way analysis of variance (ANOVA), followed Bonferroni test. Significance for results was set at P < 0.05.

## Additional Information

**How to cite this article**: Fujita, Y. *et al.* Supplementation with D-serine prevents the onset of cognitive deficits in adult offspring after maternal immune activation. *Sci. Rep.* **6**, 37261; doi: 10.1038/srep37261 (2016).

**Publisher’s note:** Springer Nature remains neutral with regard to jurisdictional claims in published maps and institutional affiliations.

## Figures and Tables

**Table 1:** Levels of amino acids and their ratios in the frontal cortex, hippocampus and striatum of the juvenile offspring after MIA.

	Glutamate	Glutamine	Glycine	L-Serine	D-Serine	GABA
Frontal cortex						
Control	9.882 ± 0.141	5.446 ± 0.133	0.665 ± 0.011	0.674 ± 0.015	0.357 ± 0.008	2.683 ± 0.079
Poly(I:C)	10.713 ± 0.223**	5.871 ± 0.075**	0.724 ± 0.025	0.727 ± 0.023	0.367 ± 0.011	1.991 ± 0.035***
Hippocampus						
Control	8.905 ± 0.153	5.433 ± 0.083	0.712 ± 0.012	0.656 ± 0.015	0.268 ± 0.008	2.415 ± 0.057
Poly(I:C)	8.507 ± 0.120*	5.399 ± 0.090	0.835 ± 0.042*	0.687 ± 0.017	0.265 ± 0.006	2.447 ± 0.054
Striatum						
Control	8.326 ± 0.148	5.985 ± 0.131	0.739 ± 0.015	0.666 ± 0.018	0.282 ± 0.007	2.659 ± 0.110
Poly(I:C)	8.257 ± 0.176	6.055 ± 0.104	0.883 ± 0.053*	0.745 ± 0.022*	0.296 ± 0.006	2.742 ± 0.061
	**Glutamine/Glutamate**	**L-Serine/Glycine**	**D-Serine/L-Serine**	**GABA/Glutamate**		
Frontal cortex						
Control	1.825 ± 0.041	1.016 ± 0.025	0.530 ± 0.007	0.272 ± 0.007		
Poly(I:C)	1.824 ± 0.027	1.012 ± 0.027	0.506 ± 0.005**	0.187 ± 0.005***		
Hippocampus						
Control	1.640 ± 0.022	0.925 ± 0.024	0.409 ± 0.011	0.271 ± 0.005		
Poly(I:C)	1.578 ± 0.014*	0.851 ± 0.033	0.386 ± 0.006	0.288 ± 0.005*		
Striatum						
Control	1.396 ± 0.030	0.905 ± 0.026	0.424 ± 0.007	0.320 ± 0.014		
Poly(I:C)	1.363 ± 0.017	0.872 ± 0.031	0.400 ± 0.006**	0.334 ± 0.009		

Data (nmol/mg tissue) are expressed as the mean ± SEM (Control: n = 13, Poly(I:C): n = 19). *P < 0.05, **P < 0.01, ***P < 0.001 compared to control group (Student’s t test).

**Table 2:** Levels of amino acids and their ratios in the frontal cortex, hippocampus and striatum of the adult offspring after MIA.

	Glutamate	Glutamine	Glycine	L-Serine	D-Serine	GABA
Frontal cortex						
Control	9.394 ± 0.201	5.114 ± 0.162	0.728 ± 0.022	0.742 ± 0.022	0.370 ± 0.011	2.492± 0.050
Poly(I:C)	8.953 ± 0.140	4.777 ± 0.111	0.722 ± 0.015	0.697 ± 0.020	0.333 ± 0.009*	2.544 ± 0.062
Hippocampus						
Control	9.816 ± 0.147	5.165 ± 0.113	0.909 ± 0.016	0.860 ± 0.018	0.354 ± 0.009	2.562 ± 0.054
Poly(I:C)	9.361 ± 0.134*	4.911 ± 0.094	0.967 ± 0.026	0.735 ± 0.014***	0.284 ± 0.007***	2.675 ± 0.071
Striatum						
Control	8.128 ± 0.189	5.537 ± 0.154	0.921 ± 0.029	0.815± 0.022	0.320 ± 0.009	2.973 ± 0.093
Poly(I:C)	8.562 ± 0.172	5.636 ± 0.127	1.015 ± 0.025*	0.745 ± 0.012*	0.288 ± 0.007**	3.182 ± 0.101
	**Glutamine/Glutamate**	**L-Serine/Glycine**	**D-Seri/L-Serinene**	**GABA/Glutamate**		
Frontal cortex						
Control	1.848 ± 0.043	1.027 ± 0.037	0.499 ± 0.005	0.267 ± 0.008		
Poly(I:C)	1.883 ± 0.040	0.967 ± 0.021	0.478 ± 0.007*	0.285 ± 0.007		
Hippocampus						
Control	1.909 ± 0.043	0.949 ± 0.020	0.412 ± 0.008	0.262 ± 0.006		
Poly(I:C)	1.911 ± 0.031	0.765 ± 0.021***	0.387 ± 0.007*	0.286 ± 0.006*		
Striatum						
Control	1.475 ± 0.033	0.892 ± 0.027	0.394 ± 0.009	0.369 ± 0.016		
Poly(I:C)	1.523 ± 0.026	0.738 ± 0.016***	0.386 ± 0.004	0.373 ± 0.013		

Data (nmol/mg tissue) are expressed as the mean ± SEM (Control: n = 13, Poly(I:C): n = 13). *P < 0.05, **P < 0.01, ***P < 0.001 compared to control group (Student’s t test).
